# Aspirin inhibits the SHH/GLI1 signaling pathway and sensitizes malignant glioma cells to temozolomide therapy

**DOI:** 10.18632/aging.101224

**Published:** 2017-04-17

**Authors:** Jianguang Ming, Bo Sun, Ziwei Li, Lin Lin, Xiangqi Meng, Bo Han, Ruijia Wang, Pengfei Wu, Jianlong Li, Jinquan Cai, Chuanlu Jiang

**Affiliations:** ^1^ Department of Neurosurgery, The Second Affiliated Hospital of Harbin Medical University, Harbin 150086, China; ^2^ Chinese Glioma Cooperative Group (CGCG), Beijing 100050, China; ^3^ Neuroscience Institute, Heilongjiang Academy of Medical Sciences, Harbin 150086, China

**Keywords:** aspirin, glioma, hedgehog, temozolomide, DNA damage

## Abstract

Aberrant activation of sonic hedgehog (SHH)/glioma-associated oncogene homolog 1 (GLI1) pathway plays an important role in the tumorigenicity of malignant glioma cells and resistance to temozolomide (TMZ). Here we investigated the aspirin's antineoplastic molecular route by targeting SHH/GLI1 pathway and examined the feasibility of aspirin combined with TMZ therapy. Western blot and quantitative real-time polymerase chain reaction (qRT-PCR) revealed that the activity of the SHH/GLI1 pathway was strongly inhibited by aspirin. Aspirin acted as the glioma growth-inhibitory and pro-apoptosis roles by inhibiting the SHH/GLI1 pathway and reprogramming the epithelial to mesenchymal transition (EMT). The immunofluorescence assay showed aspirin could prevent the nuclear translocation of GLI1 to inhibit its transcriptional regulation. The stable lentiviral overexpression of GLI1 reversed the DNA double strand breaks (DSBs) caused by the GANT61 and TMZ. Furthermore, aspirin combined with TMZ enhanced chemosensitivity and GLI1-induced chemoprotection was partly blocked by aspirin *in vitro* and *in vivo*. Collectively, aspirin has a therapeutic potential for SHH/GLI1 targeted therapy against glioma cells. Acquired activation of GLI1 protects glioma cells against TMZ therapy. Impairment of DNA DSBs repair activity might be involved in the route of aspirin-induced chemosensitivity. Combined aspirin with TMZ may be a promising strategy against malignant glioma.

## INTRODUCTION

Glioma accounts for 45.2% of primary malignant brain tumors, which is the most aggressive, incurable and frequent brain cancer in adults. Despite of the advances of the therapeutic mainstay made in maximal surgery, combined radiation and chemotherapy (alkylating agent temozolomide), the median malignant glioma patients' survival has remained at a poor 12-16 months and the 5-year survival rate is less than 10% [1]. Glioma poses heterogeneous characteristics, challenging to current treatments for the disease. After conventional therapy, residual cells display mesenchymal and tumor-initiating features and could exhibit drug resistance and local recurrence. Thus, new strategies to overcome glioma are urgently needed.

The Hedgehog signaling pathway is a key regulator of the central nervous system during embryogenesis, which is often up-regulated in many cancers like glioma and plays a role in both tumorigenesis and progression. SHH activates a signal transduction, leading to the activity of GLI1, which is the reliable marker of pathway activity. It regulates transcription of downstream oncogenes, such as Foxm1, Bcl-2 and N-myc, which played important roles on cell survival, invasion and cancer stem cell self-renewal [2]. In addition, it was indispensable for EMT and chemoresistance [3]. The SHH/GLI1 signaling had also been reported to be activated by different non-canonical pathways including the NF-κB pathway, which could be regulated by the nonsteroidal anti-inflammatory drug aspirin [4-6]. So, we assessed whether aspirin could be an effective agent in the treatment of malignant glioma via inhibiting the SHH/GLI1 signaling pathway pharmacologically.

Temozolomide is a critical medication therapy for patients with glioma. TMZ exerts its effects mainly via the mutagenic product O^6^-methylguanine, a cytotoxic DNA lesion leading to DNA base mismatch repair, which results in DSBs and cell apoptosis, but the ultimate efficacy of TMZ is variable according to intrinsic and acquired TMZ-resistance. The expression of O^6^-methylguanine-DNA methyltransferase (MGMT) has always been linked to the intrinsic drug resistance, which can transfer the alkyl/methyl adducts to its own cysteine residues. The MGMT molecule is irreversibly inactivated, so the protein synthesis determines the repair capability of MGMT [7]. Moreover, some studies demonstrated GLI1 inhibition induced almost all DNA damage in human cancer, which suggested acquired overexpression GLI1 could induce non-canonical TMZ-resistance in glioma cells [8].

Aspirin (acetylsalicylic acid, ASA) is the most widely used drug for its analgesic, antipyretic, and antiinflammatory properties. However, aspirin has a broadspectrum antitumor effect in various tumor cells, such as pancreatic cancer, colorectal adenomas, hepatocellular carcinoma, etc. [9-11], yet poorly understood in glioma. Epidemiological evidence and previous experimental data have shown that regular long-term aspirin use positively correlated with risk for glioma, although this finding was not reported consistently resulting from selection biases and other factors of the epidemiological studies [12]. Also, aspirin has been reported to prevent and treat cancer metastasis effectively and safely [13]. Here we confirm that aspirin exerts antineoplastic property in glioma by abrogating the tumorigenic effect of the SHH/GLI1 signaling pathway and acquire in-depth knowledge of sensitizing route of aspirin to TMZ therapy.

## RESULTS

### Effects of aspirin on glioma cells proliferation

**Figure 1:**
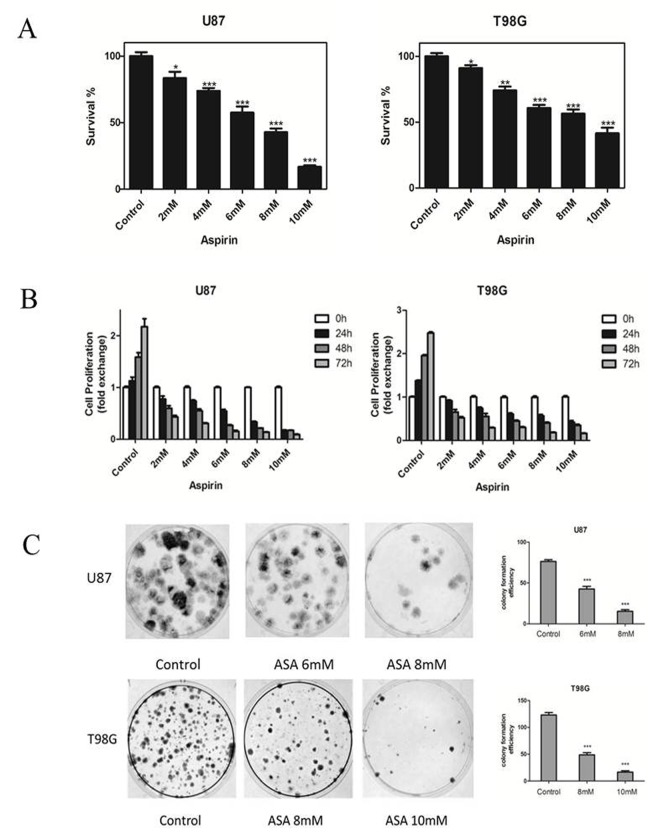
Effects of aspirin on glioma cells survival and proliferation (**A**) U87 and T98G cells were treated with increasing concentrations of aspirin for 24 h. Cell survival was analyzed by CCK-8 assay. (**B**) U87 and T98G cells were treated with indicated concentrations of aspirin and harvested after 24, 48 and 72 h. Cell proliferation was analyzed by CCK-8 assay. (**C**) The glioma cells viability upon indicated concentrations of aspirin treatment were determined by colony formation assay. Data are shown as mean±SEM for the three replicates. Statistical significance levels are indicated as: *P < 0.05; **P < 0.01 and ***P < 0.001.

We first tested the effects of aspirin on the two glioma cells growth. The number of the cells decreased as measured by CCK-8 after 24 h treatment with aspirin compared with DMSO as control (Fig. 1A). The sensitivities to aspirin between the two glioma cell lines were different. The medial IC50 was 6.32 mM for U87 and 8.45 mM for T98G respectively. These results showed that aspirin was cytotoxic against glioma cells in a dose-dependent manner. Therefore, we separately chose 6 mM, 8 mM for U87 and 8 mM, 10 mM for T98G in the subsequent study. Next, we determined the cytostatic effect of aspirin on U87 and T98G cells. The two cell lines were exposed to graded doses of aspirin (2, 4, 6, 8, 10 mM) for different time intervals (0, 24, 48 and 72 h). Both cell lines showed a time-dependent manner with the aspirin treatment (Fig. 1B). Similarly, aspirin treatment also decreased the colony numbers in the two cell lines (Fig. 1C). These results indicated that the aspirin's inhibition effect for glioma cells was dose-dependent and cumulative over time.

### Restrained effects of aspirin treatment on the invasion, migration of glioma cells and the expression of EMT-related marker

**Figure 2:**
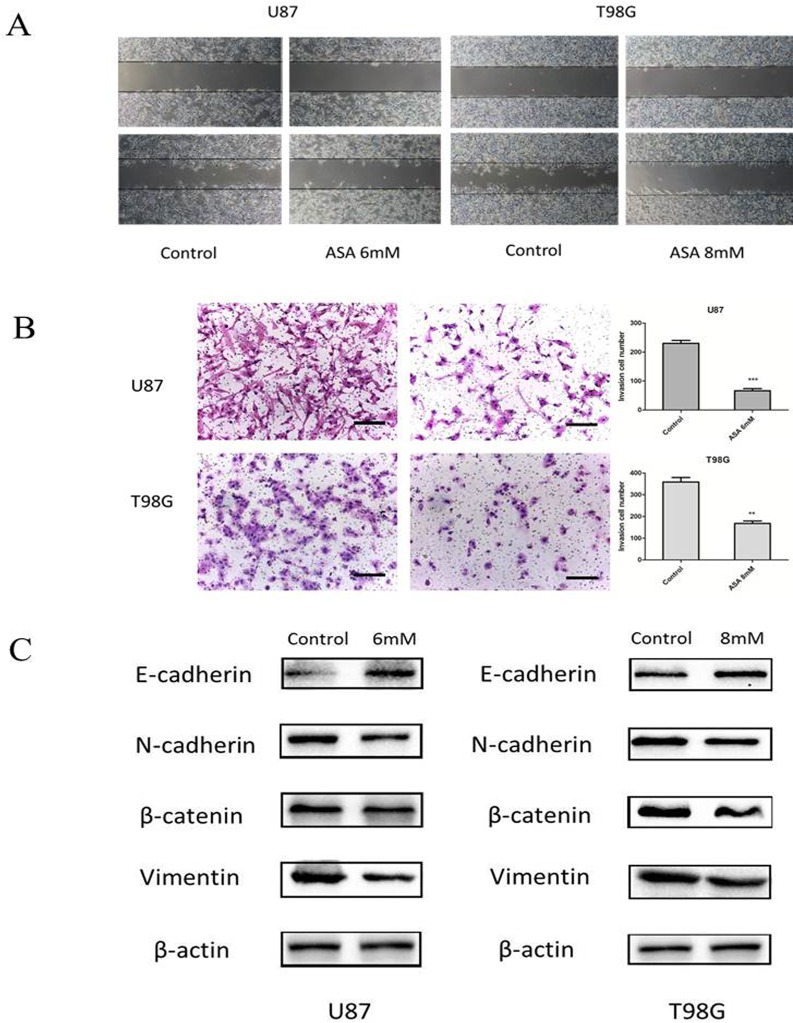
Aspirin treatment on the invasive property of glioma cells (**A**) U87 and T98G cells migration were analyzed using the scratch-wound assay. (**B**) U87 and T98G cells invasive ability were analyzed by the transwell assay after 24 h of treatment with indicated concentrations of aspirin. Bars represent 50 μm. (**C**) Western blot analysis showed that expression of EMT-related markers was altered by aspirin treatment. β-actin was used as an internal control. Data are shown as mean±SEM for the three replicates. Statistical significance levels are indicated as: *P < 0.05; **P < 0.01 and ***P < 0.001.

Glioma cells presented highly invasive characteristic *in vitro* and *in vivo*. To investigate the role of aspirin in glioma cells migration and invasion, scratch-wound assay and transwell assay were performed. The assay revealed that aspirin repressed the migration and invasion capacity of U87 and T98G cells compared with DMSO as control (Fig. 2A-B). To further investigate the related markers of invasion, we analyzed the expression of N-cadherin, β-catenin, Vimentin and E-cadherin in U87 and T98G cells. We observed that aspirin decreased the levels of β-catenin, Vimentin, N-cadherin and increased E-cadherin (Fig. 2C), indicating that aspirin could inhibit the invasion and migration capacity via repressing the EMT.

### Aspirin induces apoptosis

**Figure 3:**
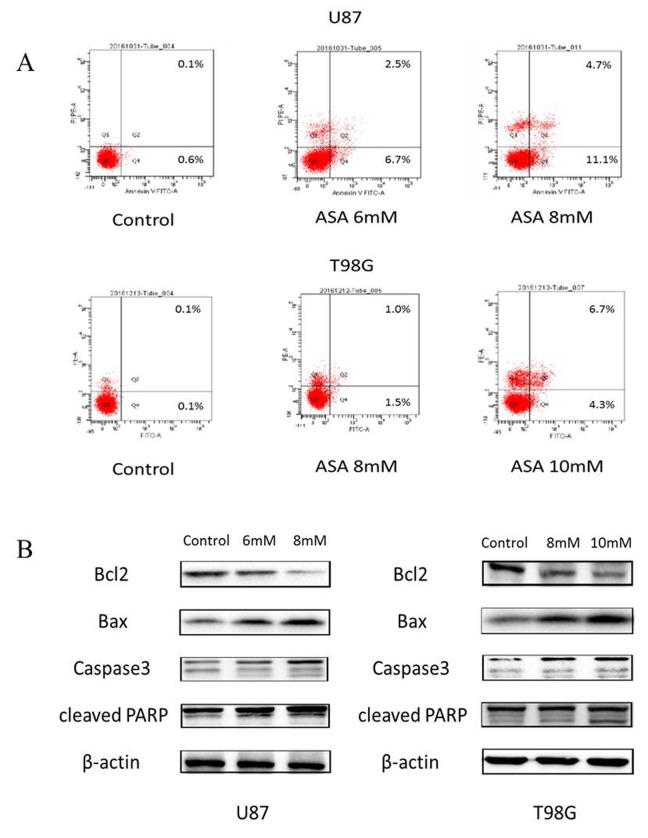
Aspirin induced apoptosis through the internal pathway in glioma cells (**A**) The U87 and T98G cells were treated with indicated concentrations of aspirin for 24 h, and the promotion of apoptosis was measured by AnnexinV-FITC/PI double staining. A representative experiment of three replicated assays was shown. (**B**) Western blot analysis showed Bcl-2, Bax, Caspase 3 and cleaved PARP proteins expression in U87 and T98G cells treated with the indicated concentrations of aspirin for 24h. β-actin was used as an internal control.

As the anti-apoptosis correlated with the aberrant Hh activation [14] and the GLI1 target genes (Foxm1 and Bcl-2) bound up with cell survival [15], we adopted the Annexin V/PI staining to verify whether aspirin could induce apoptosis. The result revealed that aspirin induced the apoptosis of both U87 and T98G cells in a dose-dependent (Fig. 3A). Subsequently, to investigate the aspirin associated apoptotic route via SHH/GLI1 inhibition at protein level, Western blot showed that Caspase-3, Bax and cleaved PARP increased in a dosedependent manner and that Bcl-2, an anti-apoptosis protein, decreased in the glioma cells treated by aspirin (Fig. 3B). Together, these data suggested that aspirin could induce apoptosis.

### Aspirin inhibits SHH/GLI1 signaling in glioma cells

**Figure 4:**
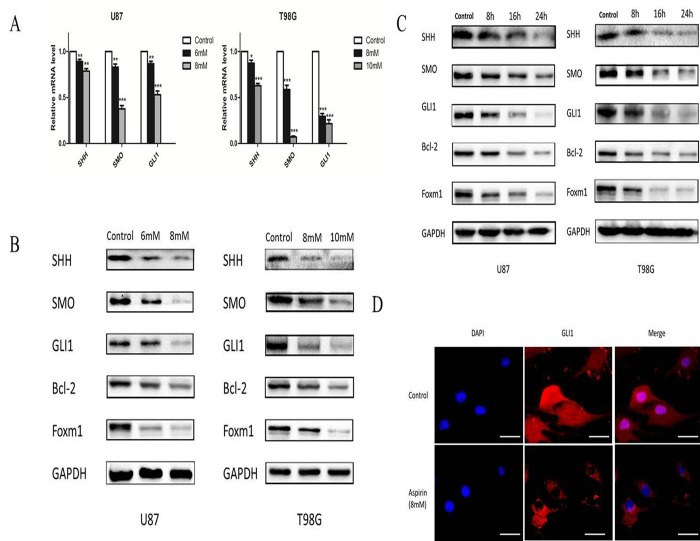
Aspirin inhibited SHH/GLI1 signaling pathway (**A**) QRT-PCR analysis of SHH, SMO, and GLI1 mRNA from U87 and T98G cells following indicated concentrations of aspirin treatment for 24 h. (**B**) Western blot analysis showed SHH, SMO, GLI1, Bcl-2 and Foxm1 proteins expression in U87 and T98G cells treated with indicated concentrations of aspirin for 24 h. GAPDH was used as an internal control. (**C**) Western blot analysis showed SHH, SMO, GLI1, Bcl-2 and Foxm1 proteins expression in U87 and T98G cells treated with the indicated concentrations of aspirin (8 mM for U87, 10 mM for T98G) for different time intervals (8, 16 and 24 h). GAPDH was used as an internal control. (**D**) U87 cells treated with 8mM aspirin for 24 h were subjected to immunofluorescent assay using anti-GLI1 antibody. Data are shown as mean±SEM for the three replicates. Statistical significance levels are indicated as: *P < 0.05; **P < 0.01 and ***P < 0.001.

SHH/GLI1 signaling pathway was important to maintain the growth invasion and anti-apoptosis of glioma cell [16]. We hypothesized that aspirin may abrogate the activity of GLI1 to inhibit the glioma cells malignancy. U87 and T98G cells were treated with different doses of aspirin for 24 h and qRT-PCR assay revealed that SHH, SMO and GLI1 mRNA expression decreased in a dose-dependent manner (Fig. 4A).

Western blot also verified aspirin could downregulate SHH, SMO and GLI1 proteins expression in a time- and dose-dependent manner (Fig. 4B-C). To further confirm the hypothesis, the expression of GLI1 target genes (Foxm1 and Bcl-2), essential for cell growth and survival, were apparently attenuated after aspirin treatment (Fig. 4B-C). In addition, immunofluorescent assay revealed that aspirin prevented most of GLI1 translocation into nucleus and resulted in lower expression level at the cytoplasma (Fig. 4D). These data revealed that aspirin could inhibit SHH/GLI1 signaling in glioma cells.

### GLI1 induces non-canonical TMZ-resistance in glioma cells

**Figure 5:**
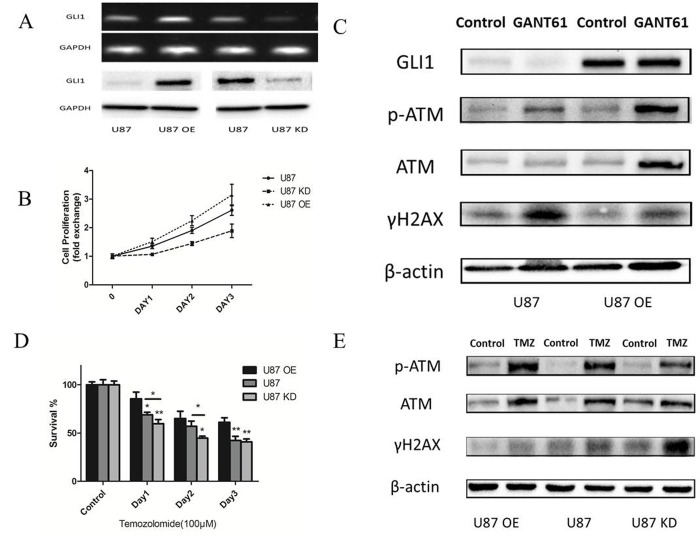
GLI1 acted as an effector inducing non-canonical TMZ-resistance in glioma cells (**A**) Relative expression of GLI1 in U87, U87 OE and U87 KD. (**B**) Effect of GLI1 on cell proliferation was evaluated by CCK8 assays in U87, U87 OE and U87 KD cells. (**C**) Western blot analysis showed GLI1, γH2AX, p-ATM and ATM proteins expression in U87 and U87 OE treated with GANT61 (5 μM ) for 48 h. β-actin was used as an internal control. (**D**) U87, U87 OE and U87 KD were examined for their sensitivity to TMZ at concentration of 100 μM. (**E**) Western blot analysis showed γH2AX, p-ATM and ATM proteins expression in U87, U87 OE and U87 KD treated with TMZ. β-actin was used as an internal control. Data are shown as mean±SEM for the three replicates. Statistical significance levels are indicated as: *P < 0.05; **P < 0.01 and ***P < 0.001.

After we built the U87 stably overexpressing GLI1 (U87 OE) and U87 knowdown GLI1 (U87 KD) with the recombinant lentivirus construct, PCR and Western Blot were performed to verify the GLI1 expression (Fig. 5A). CCK-8 assay revealed that GLI1 overexpression could promote the growth of U87 cells and that the GLI1 knowdown could inhibit the growth of U87 cells (Fig. 5B). Then we used the GANT61 (5 μM), an inhibitor of the GLI1, to abrogate the GLI1 expression in the U87 OE and U87 and tested some DNA damage repair markers by the Western blot 48 h later. Our results showed that U87 OE had more p-ATM and less γH2AX compared with the U87, indicating that the overexpression GLI1 could strengthen DNA damage repair capacity and reduce the DSBs (Fig. 5C). Then, to determine the effect of GLI1 on cell sensitivity to TMZ, we treated U87, U87 OE and U87 KD cells with TMZ (100 μM) for 3 days. The overexpressed GLI1 increased the resistance against TMZ therapy as compared to control. On the other hand, the knockdown GLI1 enhanced the sensitivity to TMZ therapy as compared to control (Fig. 5D). Also, the Western blot analysis showed that the overexpressed GLI1 enhanced p-ATM and reduced γH2AX caused by TMZ therapy (Fig. 5E). The results displayed that GLI1 dependence manner for DNA damage repair activity could mediate non-canonical TMZ-resistance in glioma cells.

### Aspirin overcomes intrinsic and non-canonical TMZ-resistance

**Figure 6:**
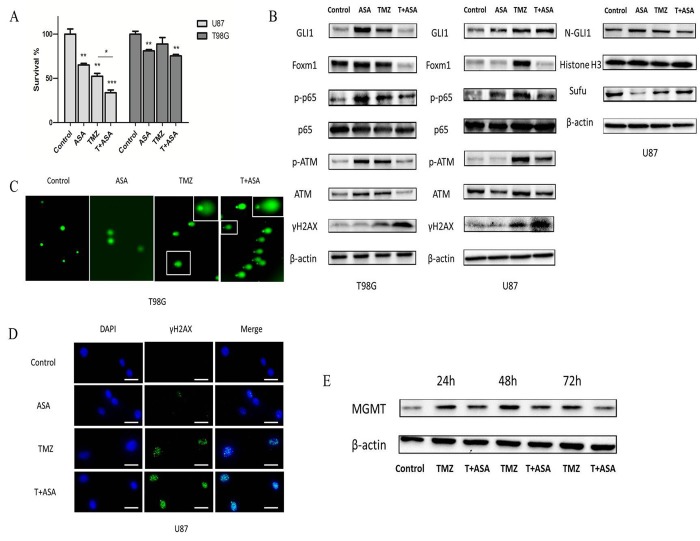
Aspirin sensitized TMZ therapy (**A**) U87 and T98G cells were treated with aspirin (1 mM) alone, TMZ (100 μM) alone or both for 72 h. Cell survival was analyzed using CCK-8 assay. (**B**) U87 and T98G cells were treated by aspirin (1 mM) alone, TMZ (100 μM) alone or both for 72 h. Western blot analysis showed GLI1, Foxm1, p-p65, p65, γH2AX, p-ATM, ATM, N-GLI1 and Sufu proteins expression. β-actin and Histone H3 were used as an internal control. T98G and U87 cells were treated with aspirin (1 mM) alone, TMZ (100 μM) alone or both for 72 h and then collected for comet assay (**C**) and immunofluorescence (**D**) respectively. (E) TMZ-induced upregulation of MGMT in T98G cells after exposed to the TMZ (100 μM) with or without aspirin (1 mM) were assayed at the end of each time-point by Western blot. Data are shown as mean±SEM for the three replicates. Statistical significance levels are indicated as: *P < 0.05; **P < 0.01 and ***P < 0.001.

We chose 1 mM aspirin as ‘therapeutic levels’ in the subsequently study which also could support further clinical translation [16]. Therapeutic levels of aspirin have already been reported to counteract the pro-tumorigenic effects of the IL-6 by slowing down the ribosome biogenesis rate [17]. In our study, to examine the effect of TMZ with or without aspirin treatment on glioma cell growth, TMZ and aspirin at concentrations of 100 μM and 1 mM respectively were adopted and then measured cell proliferation in 3 days. Obviously, aspirin further primed the anti-neoplastic efficacy of TMZ (Fig. 6A). In addition, the Western blot analysis showed that the γH2AX of the combined group was more prominent than that in the TMZ alone group (Fig. 6B). At the same time, we used comet assay and γH2AX foci immunofluorescence to reexamine the levels of the DNA damage in T98G and U87 cells respectively. Compared to group treated with TMZ or aspirin alone, longer tail (tail intensity, marker for the degree of DNA damage) and the higher γH2AX expression level showed that the combined group significantly induced DNA damage (Fig. 6C-D). Compared with TMZ alone, the combined group inhibited the p-ATM and p-NF-κB (p-p65) expression. Similarly, the combined group reduced the total GLI1 of the T98G and the nuclear GLI1 (N-GLI1) of the U87 which was consistent with the p-ATM and p-p65 protein trends. The combined group could increase the Sufu expression than the TMZ alone group, which could inhibite GLI1 nuclear localization and promote GLI1 proteolysis in the U87 cells as we have reported before [18]. Also, the Foxm1 was reduced in the combined group (Fig. 6B). Collectively, data demonstrated that aspirin repressed the GLI1 activation following TMZ treatment in the combined group, which overcame non-canonical TMZ-resistance in glioma cells.

In addition, MGMT transcription could be induced by TMZ [19] and it has been reported that GLI1 potentially regulated the transcriptional activity of MGMT gene [20]. In our study, we investigated whether the MGMT expression could be affected in T98G cells exposed to TMZ combined aspirin. T98G cells were incubated in culture medium containing TMZ (100 μM) with or without aspirin (1 mM). Drug removal after 3 h allowed culture recovery and then assayed for MGMT expression after 24 h, 48 h and 72 h. The TMZ alone notably induced the upregulation of the MGMT protein expression, while the aspirin in the combined group could reverse it (Fig. 6E), indicating that the combined treatment could suppress the *de novo* synthesis of MGMT protein. Our results suggested that aspirin could overcome the intrinsic TMZ-resistance.

### Verification of aspirin sensitizing TMZ treatment through SHH/GLI1 signaling in vivo

**Figure 7:**
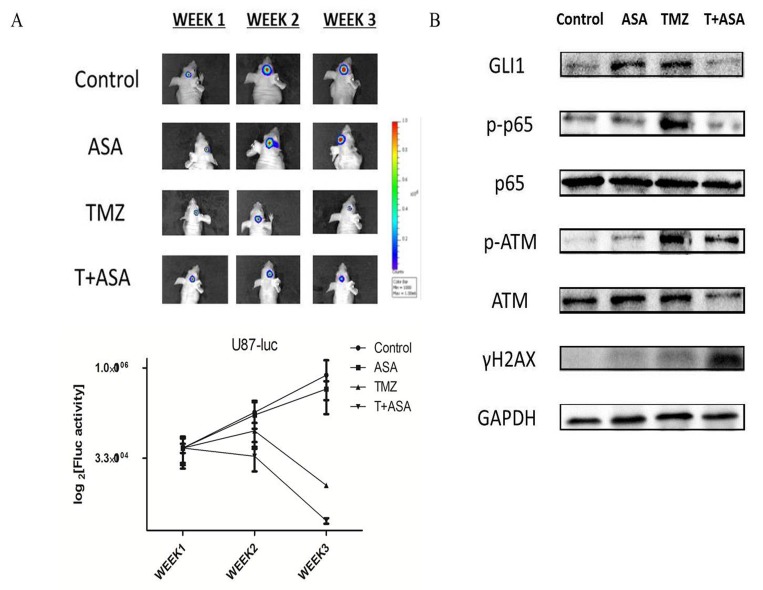
Verification of aspirin sensitizing TMZ treatment through SHH/GLI1 Signaling *in vivo* (**A**) Representative luminescence imaging and mean Fluc activity values of different treatment groups every week. (**B**) The subcutaneous U87 xenografts tissue were removed and subjected to Western blot analysis of GLI1, p-p65, p65, γH2AX, p-ATM and ATM. GAPDH was used as an internal control.

To verify that aspirin could potentiate TMZ treatment and the molecular route, we used orthotropic and subcutaneous xenografts to establish nude mice model. We assessed tumor growth every week by bioluminescence imaging. Fluc activity demonstrated the ASA slightly reduced the tumor growth and the combined treatment could significantly reduce tumor growth compared to the TMZ alone (Fig. 7A). Then we collected the tumor tissue from the mice model and tested some molecular alteration. The results also showed that TMZ combined with aspirin could significantly reduce the level of GLI1, p-p65 and p-ATM and increased the γH2AX (Fig. 7B). Our study revealed that aspirin could enhance chemosensitivity in a preclinical mice model *in vivo*.

## DISCUSSION

Glioblastoma (GBM) poses aggressive and heterogeneous properties, challenging to multimodal treatment with surgery, radiotherapy and temozolomide therapy. Although the clinical use of TMZ represents an improvement in the treatment of GBM, the drug resistance reduces patient survival and carries a poor prognosis. Deep understanding of the molecular biology of GBM accelerates innovative clinical trials [21]. Aberrant activation of SHH/GLI pathway in the adult leads to cellular proliferation manifests as cancer [22]. Also, with target gene products it could contribute to the functional properties of cancer cells, such as survival, migration, and neoangiogenesis [23]. The GLI1, reliable molecular marker of the SHH/GLI1 pathway activity, is essential to GBM chemoresistance [24]. Inhibition of SHH/GLI1 signaling pathway restricted key cellular processes, such as cell cycle, EMT and metabolism [25, 26]. So, targeting the GLI1 might become a novel and potential therapeutic strategy for glioma. However, many tools such as siRNA and specific inhibitors have been limited due to drug resistance and potent side effects. In contrast, epidemiologic studies indicated that daily use of ASA was associated with reduced risk in various cancers including gliomas and reduced longterm risk of death due to cancer too [12, 27, 28]. The mechanism through which daily aspirin works was about reducing cell proliferation by inhibiting ability of platelets' implications for the oncoprotein c-MYC [29]. Furthermore, carcinogenic inflammation has become a major factor in the pathogenesis of human cancer, which regulates malignant cells CSC features, angiogenesis, metastasis and alters chemo- and radio-sensitivity. Aspirin could inhibit the cancer-related inflammation which has been identified as new target to improve treatment effect [10, 17]. Also, it was argued that aspirin exerted antiglioma effect by inhibiting myeloid-derived suppressor cells, NF-κB, β-catenin, IL-6-STAT3 signaling pathway [30-32]. But, the route of aspirin's antineoplasm is not clear. So, it needs further study to demonstrate the effect of aspirin on glioma. Firstly, our study found that aspirin could inhibit the SHH/GLI1 signaling pathway with a coordinated decrease in SHH, SMO, GLI1 and GLI1 targets expression. Considering that GLI1, a crucial trans-cription factor, played important roles in the glioma cell phenotype, we verified that aspirin reduced cell growth in a dose- and time-dependent manner. Aspirin also regulated EMT to inhibit the invasion and migration of glioma cells. Meanwhile, the Foxm1 gene downstream of GLI1, via targeting CyclinD1 and MMP-2 and regulating TGF-β1 signaling, is critical in promoting EMT of glioma cells [33]. In the current study, we supposed that aspirin could downregulate Foxm1 to partially inhibit the invasion. Moreover, we confirmed that aspirin promoted glioma cells apoptosis. The antiapoptotic Bcl-2 decreased in aspirin-treated cells, which was involved in internal apoptosis pathway. So, we continued to examine the expression of Bax, Caspase-3 and cleaved PARP. Our results illustrated that aspirin could promote the apoptosis of glioma cells. Aspirin alone was used to treat glioma cell lines, and that concentrations of aspirin from 1 to 10 mM inhibited the SHH/GLI1 signaling pathway significantly. Although aspirin was a fat-soluble molecule which could cross the blood-brain barrier, the high dose in our experiment may lead to gastrointestinal bleeding, tinnitus, renal failure and coma if applied clinically. Nanoparticle research could contribute to clinical application of aspirin in glioma patients, which can encapsulate aspirin and deliver it directly to the glioma cells instead of via the blood circulation system, which would significantly reduce side effects [34, 35].

Next, we analyzed whether inhibition of GLI1 influenced DNA damage and repair in the glioma cells. The GLI1 inhibitor GANT61 could induced the γH2AX, a good recognized marker of DNA DSBs in U87 cells. In contrast, γH2AX was slightly detectable in U87 OE cells. Additionally, the GLI1 overexpression might increase more DNA repair activity by regulating the phosphorylation of ATM. Therefore, we observed that GANT61 impacted the DNA DSBs by inhibiting the GLI1 expression and that GLI1 overexpression could enhance the DSBs repair activity. It was known that GLI1 imparted drug resistance through inducible glucuronidation in the Acute Myeloid Leukemia [36]. Meanwhile, GLI1 contributed to cellular resistance through altering cellular drug accumulation in the ovarian cancer [37]. The results displayed that GLI1 dependence manner for DNA damage repair activity could mediate non-canonical TMZ-resistance in glioma cells.

Subsequently, our study revealed that aspirin could abrogate TMZ-induced activation of GLI1 *in vitro* and *in vivo*. Because we found short time of aspirin administration promoted GLI1 expression *in vitro* and *in vivo*, low-dose of aspirin with limited side effects was repurposed to overcome TMZ resistance of glioma. Since the aspirin dose was therapeutic level with limited cytotoxicity, the cells were rescued by DNA damage repair system before 72 h, expressing increased GLI1 in feedback. May DNA damage repair activity enhanced by GLI1 overexpression maintained the cell survival after low dose asprin treatment. Compared with U87 cells, T98G responsively expressed more GLI1, p-p65 and p-ATM fold change which contributed to the cell survival and antiapoptosis in the aspirin treatment. In the combined treatment, the TMZ induced p-p65 could be significantly reduced by the aspirin which further inhibited the total or nuclear GLI1 expression as well as p-ATM and increased the γH2AX *in vitro* and *in vivo*. In addition, the aspirin delayed the MGMT synthesis; equals it could enhance the enzyme depletion induced by TMZ.

SHH/GLI1 signaling pathway has been characterized as a NF-κB target pathway that promotes NF-κB-mediated apoptosis resistance in cancers [38]. Given that NF-κB is known to be activated by genotoxic agent, alkylating agents [39, 40], which might play a role in the acquired overexpressed GLI1 from glioma cells responding to TMZ therapy. Also, DSBs responsive ATM cascade contributed to the initiation of NF-κB activation as suggested previously [41, 42]. So, TMZ-induced NF-κB activated GLI1 and GLI1-upregulated DNA repair activity involving activation of the ATM that contributed to ATM-dependent NF-κB activation in feedback, suggesting that acquired GLI1 activation usurped a route that was important to initiate cycle cascade leading to TMZ resistance. TMZ combined with aspirin disturbed the NF-κB -GLI1-ATM feedback loop. Our results suggested that aspirin exhibited chemosentivity through inhibiting GLI1 to impair the repair activity following TMZ and that less ATM activation alleviated NF-κB pathway in feedback.

In conclusion, our studies revealed that aspirin could inhibit glioma cells proliferation, induce apoptosis through SHH/GLI1 signaling pathway and aspirin potentially attenuate intrinsic and non-canonical TMZ resistance. Our studies provided the experimental evidence and support for the usage of aspirin to enhance sensitivity fo TMZ for glioma.

## MATERIALS AND METHODS

### Reagents

Aspirin (acetylsalicylic acid, ASA) and Temozolomide were obtained from Sigma-Aldrich (St Louise, USA). GANT61 was obtained from Selleck Chemical (Houston, USA). Cell culture medium (Dulbecco's modified Eagle's medium) and fetal bovine serum were purchased from Biological Industries (Bioind).

### Cell lines culture and lentiviral transduction

The human glioma cell lines, U87 and T98G, were purchased from Chinese Academy of Sciences Cell Bank. U87 cells overexpressing GLI1 (U87 OE, 140125DZ) and knockdown GLI1 (U87 KD, 140103AZ) were transfected with recombinant lentivirus construct purchased from GenePharma (Shanghai, China) according to the manufacturer's instructions. Transduction and selection of stable cells were performed as described before [43]. The stable transfected cells were testified by PCR and Western blot analysis and used for further research.

### Cell survival assay

The glioma cells growth was evaluated using the Cell counting kit-8 (CCK-8) assay according to the manufacturer's instructions. Briefly, cells were seeded at density of 3000-6000 cells/well in a 96-well plate and incubated overnight. Cells were treated with fresh medium containing various concentrations of aspirin for 24 h. Subsequently, CCK-8 solution was added and the viable cells were quantified using IMARK microplate reader at 450 nm of absorbance.

### Cell proliferation assay

The CCK-8 assay was used to test relative cell growth for different time intervals (0, 24, 48 and 72 h) as above. Each experiment was performed in triplicate. All cell proliferation assays were repeated as independent experiments for three times.

### Colony formation assay

Briefly, glioma cells were seeded into wells of a 6-well plate in the amount of 500 cells per well for 24 h. The cells were treated with or without indicated concentrations of aspirin and the culture medium containing aspirin was removed after 24 h. Then the cells were washed with phosphate-buffered saline (PBS) and cultured for 2 weeks to allow colony formation. The culture medium was changed every 2 days. The obtained colonies were washed with PBS and fixed in methanol for 25 min at room temperature and stained with Giemsa stain. Subsequently, the number of macroscopically observable colonies were counted and compared with negative control group. Each experiment was performed in triplicate.

### Migration (scratch-wound) assay and invasion assay

U87 and T98G cells were seeded into the 6-well plates until more than 70 % of confluent growth. The cells were manually scratched with a sterile pipette tip, washed with PBS, and replaced with serum-free culture medium. Photographs of the scratch-wound gap were taken at 0 and 24 h by an Axiovert 200 microscope (Carl Zeiss). For each well, at least three different fields along the scratch were analyzed in triplicate. Invasion assays were done in a 24-well Transwell chamber (Corning). Briefly, aspirin treated cells were plated in the upper chamber of Matrigel-coated well, 1 × 10^5^ cells seeding in 200 μL of serum-free medium. 500 μL medium containing 12% FBS was added to the lower part of the chamber. After incubation for 24 h, the cells on the upper surface of the membrane were removed and the filter on the lower surface of the chamber was fixed with methanol for 20 min and stained with hematoxylin-eosin for 5 and 1 min and quantified under a microscope.

### Apoptosis assay

The FITC Annexin V Apoptosis Detection Kit I (BD Pharmingen, USA) was used to detect and quantify apoptosis by flow cytometry. In brief, U87 and T98G cells in the log phase of growth reached 70–80% confluence. Aspirin was added to the medium at indicated concentrations. After 24 h, the cells were harvested and collected by centrifugation. Cells were resuspended at 1×10^6^ cells/ml with binding buffer, added 5 μL FITC Annexin V and 5μL PI and incubated for 15 min. Then the stained cells were immediately analyzed using by flow cytometry (FACSCanto II, BD Biosciences, USA).

### RNA extraction, cDNA synthesis and quantitative real-time PCR

Total RNA was isolated from cell lines using TRIzol reagent (Invitrogen, Carlsbad, USA). The cDNAs were prepared with the use of PrimeScript RT reagents Kit (TaKaRa, Dalian) as the manufacturer's protocol. QRT-PCR was performed in LightCycler 2.0 (Roche Diagnostics, CH) in triplicate and normalized with glyceraldehyde 3-phosphatedehydrogenase (GAPDH) as endogenous control. The relative expression was calculated by the 2^−ΔΔC(t)^ method. The real-time PCR primers were as follows: SHH: sense 5′-TGCTGCTAGTCCTCGTCTCCT-3′, antisense 5′-TTTTGGGGTGCCTCCTCTT-3′, SMO: sense 5′-CTTCAGCTGCCACTTCTACGACTTC- 3′, antisense: 5′-TCGGGCGATTCTTGATCTCAC-3′, GLI1: sense 5′-GAGCACGAGGGCTGCA GTAA-3′, antisense 5′-TCGCAGCGAGCTAGGATCTGTA-3′, GAPDH: sense 5′-ACGACCACTTTGTCAAGCTC-3′, antisense 5′ -GGTCTACATGGCAACTGAGA-3′.

### Immunofluorescence

Immunofluorescence assay was performed as previously described [18]. The cell lines were treated with indicated reagent or DMSO. The cells were fixed in 4% paraformaldehyde, permeabilized with 0.1% Triton, blocked with 1% BSA, and incubated with anti-GLI1 antibody (1:200, Abcam, USA), anti-γH2AX antibody (1:200, Cell Signaling Technology, USA) and Alexa Fluor 594 and 488-labeled secondary antibody (1:1000, Molecular Probes, USA). The slides were examined by fluorescence microscope (Nikon, Japan).

### Western blot analysis

After treated and untreated U87 and T98G cells lysate were harvested, equivalent total protein was separated in 7.5% (10%, 12.5%) sodium dodecyl sulfate polyacrylamide gel electrophoresis and transferred onto PVDF membrane (Millipore, USA). The membrane was blocked in 5% milk (5% BSA)–TBST solution at room temperature, and incubated separately with anti-SHH antibody, anti-SMO antibody, anti-GLI1 antibody, anti-Bcl-2 antibody, anti-Foxm1 antibody, anti-Bax antibody, anti-Caspase-3 antibody, anti-PARP antibody, anti-ATM antibody, anti-p-ATM antibody, anti-γH2AX antibody, anti-N-cadherin antibody, anti-β-catenin antibody, anti-Vimentin antibody, anti-E-cadherin antibody, anti-Histone H3 (D1H2) antibody (all from Cell Signaling Technology, USA), anti-NF-κB (p65) antibody, anti-p-NF-κB (p-p65) antibody (Wanleibio, China), anti-β-actin antibody, anti-MGMT antibody (Santa Cruz, USA) and anti-GAPDH antibody (Sigma, USA). Following incubation in HRP labeled secondary antibody (Introvigen), protein bands were scanned with the ECL system and detected by Gel Doc 2000 (Bio-Rad).

### Comet assay

The comet assay (Trevigen, Gaithersburg, MD) was performed as described previously [44]. Briefly, T98G cells were treated, trypsinized and washed with ice-cold PBS. Next, the cells at 1 × 10^5^/ml were embedded in LMAgarose and immediately pipetted 100 μL onto CometSlide. After cooling, submerse the slides flat in pre-cooled lysis buffer and freshly prepared Alkaline Unwinding Solution, pH > 13. Electrophoresis was carried out at 25 V, 300 mA. The slides were washed in neutralization buffer (0.4 M Tris-HCl, pH 7.5) for three times and in 70% ethanol. Subsequently, the DNA was stained with SYBR Green I dye (Sangon Biotech, 1:10,000 in Tris-EDTA buffer, pH 7.5) and images were captured using a fluorescence microscope (Nikon, Japan).

### Tumor xenograft study

Five-week–old female BALB/c-nude mice were used for subcutaneous and orthotropic xenograft models. U87 cells transfected with luciferase lentivirus (U87-luc 3 × 10^5^ cells in 3 μL per mouse) were intracranial injected into the right hemisphere. The U87 cells (1× 10^7^, 100 μL) were injected into left flank. Mice were observed for 1 week to ensure proper health. The second week after implantation, the mice were randomly distributed into four groups: 1) Control group (DMSO and PBS i.p.), 2) TMZ group (60 mg/kg/day in DMSO/PBS i.p.) for 5 consecutive days, 3) ASA group (orally at 50 mg/kg/day in PBS), 4) T+ ASA group (orally at 50 mg/kg/day 3 h prior to TMZ injection 60 mg/kg i.p., TMZ for 5 consecutive days only). The mice were weighed every 3 days. The intracranial tumors were measured as average radiance (photons/s/cm^2^/sr) by IVIS Lumina Imaging System (Xenogen) every week. At the end of the third week, the xenograft-bearing mice were sacrificed. The subcutaneous transplantations were for Western blot analysis to verify the molecular route *in vivo*. The aspirin dose can be considered traditional low-to-moderate. There was no evidence of GI bleeding. The study was performed following approval by the Harbin Medical University Institutional Animal Care and Use Committee.

### Statistical analysis

Statistical results with mean±SEM in the experiments were calculated using GraphPad software (version 6.0). Comparisons of each treatment data with control were assessed by the paired t-test. A value of P < 0.05 was assumed as statistical significance.

## Abbreviations

TMZ: temozolomide
qRT-PCR: quantitative real-time polymerase chain reaction
DSBs: double strand breaks
EMT: epithelial to mesenchymal transition
MGMT: O6-methylguanine-DNA methyltransferase
ASA: aspirin (acetylsalicylic acid)
CCK-8: cell counting kit-8
PBS: phosphate-buffered saline
GAPDH: glyceral-dehyde 3-phosphatedehydrogenase
U87 OE: U87 stably overexpressing GLI1 cell line
U87 KD: U87 knowdown GLI1 cell line
GBM: glioblastoma
